# Bioactive Lipids and Chronic Inflammation: Managing the Fire Within

**DOI:** 10.3389/fimmu.2018.00038

**Published:** 2018-01-29

**Authors:** Valerio Chiurchiù, Alessandro Leuti, Mauro Maccarrone

**Affiliations:** ^1^Department of Medicine, Campus Bio-Medico University of Rome, Rome, Italy; ^2^European Center for Brain Research (CERC), Santa Lucia Foundation (IRCCS), Rome, Italy

**Keywords:** eicosanoids, endocannabinoids, inflammation, resolution, specialized proresolving mediators, sphingolipids

## Abstract

Inflammation is an immune response that works as a contained fire that is pre-emptively sparked as a defensive process during infections or upon any kind of tissue insult, and that is spontaneously extinguished after elimination or termination of the damage. However, persistent and uncontrolled immune reactions act as a wildfire that promote chronic inflammation, unresolved tissue damage and, eventually, chronic diseases. A wide network of soluble mediators, among which endogenous bioactive lipids, governs all immune processes. They are secreted by basically all cells involved in inflammatory processes and constitute the crucial infrastructure that triggers, coordinates and confines inflammatory mechanisms. However, these molecules are also deeply involved in the detrimental transition from acute to chronic inflammation, be it for persistent or excessive action of pro-inflammatory lipids or for the impairment of the functions carried out by resolving ones. As a matter of fact, bioactive lipids have been linked, to date, to several chronic diseases, including rheumatoid arthritis, atherosclerosis, diabetes, cancer, inflammatory bowel disease, systemic lupus erythematosus, and multiple sclerosis. This review summarizes current knowledge on the involvement of the main classes of endogenous bioactive lipids—namely classical eicosanoids, pro-resolving lipid mediators, lysoglycerophospholipids/sphingolipids, and endocannabinoids—in the cellular and molecular mechanisms that lead to the pathogenesis of chronic disorders.

## Introduction

Inflammation represents one of the best known pathophysiological processes and represents a well-conserved mechanism evolved by vertebrates as an adaptive and defensive response to tissue injury and invasion of microorganisms that might attempt to colonize the host (1, 2). Despite the apparent simplicity of its definition, inflammation is instead a rather intricate network of cellular and molecular events, at the core of which, a plethora of pre-formed or newly synthesized mediators is elegantly arranged to obtain specific temporal and spatial responses. Endogenous lipids are arguably the most important mediators not only to be implicated in all phases of inflammation, but also to be involved in the regulation and fine-tuning of its course and cessation. Indeed, lipids are not just the major constituents of cell membranes and very efficient sources of energy, but also as key pathophysiological mediators of several intercellular and intracellular processes. Thus, during the past two decades, they have been termed “bioactive lipids,” due to their pivotal role in immune regulation, inflammation, and maintenance of tissue homeostasis (3, 4). Bioactive lipids, divided into four main families according to their biochemical functions, i.e., classical eicosanoids, specialized pro-resolving mediators (SPMs), lysoglycerophospholipids/sphingolipids and endocannabinoids (eCBs), are generated from ω-6 or ω-3 essential polyunsaturated fatty acids (PUFA) precursors, that are esterified into membrane lipids and act by binding to and activating specific G protein-coupled receptors (GPRs).

## Bioactive Lipids and Inflammation

In the event of tissue insults or infections, innate immune cells, such as granulocytes and monocytes/macrophages, are recruited to the damaged site and rapidly generate classical eicosanoids, the class of lipid mediators that is responsible for acute inflammation (or angiophlogosis) characterized by the so-called “cardinal signs” of inflammation: redness, heat, swelling, pain, and loss of function (5). Classical eicosanoids are thus highly pro-inflammatory and ignite the fire during inflammation, with the aim of removing injurious stimuli, a fire that, however, needs to be self-limiting and, eventually, promptly extinguished upon cessation or elimination of the noxious stimulus. During the last process, referred to as “resolution of inflammation” or catabasis (i.e., Dante’s descent into the hell), the very same innate immune cells recruited in the inflammatory milieu, where they produce classical eicosanoids, undergo a temporal lipid mediator class switch and start producing another class of bioactive lipids, the newly discovered SPMs. These lipids actively terminate inflammation and drive the restoration of full tissue homeostasis by activating the signs of resolution: removal, relief, restoration, regeneration, and remission (6, 7). When the fire of inflammation is not properly extinguished, due to impaired resolution, it turns into chronic inflammation (or histophlogosis), resulting in aberrant tissue remodeling and organ dysfunction (8). In this context, the outcome of inflammation depends also on the other two families of bioactive lipids, i.e., lysoglycerophospholipids/sphingolipids and eCBs, which regulate numerous cellular processes that are important for triggering those mechanisms that underlie cell and tissue adaption to inflammatory events (9, 10). Indeed, chronic inflammation represents often the causative agent and the main trigger of the damage associated to many pathologies, such as cancer, autoimmune, metabolic, cardiovascular, and neurodegenerative diseases (11, 12). Thus, it seems that bioactive lipids are largely involved in managing the fire of inflammation, either acting as fire-starters or as fire-fighters, or even as executives of the fire station.

## Classical Eicosanoids

These bioactive lipids represent probably the widest and most celebrated family, and include a huge array of molecules that have the ω-6 PUFA arachidonic acid (AA) as their common biosynthetic precursor released from membrane phospholipids by phospholipase A_2_ (13). AA is then used as a substrate for three different oxygen-incorporating enzymes that together synthesize over 120 heterogeneous and pleiotropic molecules: cyclooxygenases 1 and 2 (COX-1/2) drive the synthesis of prostaglandins (PGs), prostacyclins, and thromboxanes (TXs) (14–16), often referred together as prostanoids; 5-, 12-, and 15-lypooxygenases (5/12/15-LOX) produce leukotrienes (LTs) (17), hydroxyeicosatetraenoids (HETEs) (13), and lipoxins (LX) (6); P450 epoxygenase generates HETEs and epoxyeicosatrienoids (13). Even though all these bioactive lipids are involved in a plethora of physiological and homeostatic processes, including control of vascular tone, platelet aggregation, pain perception as well as ovulation, and embryo implantation (13, 18), they are mostly renowned for their ability to act as fire-starters and initiators of inflammation. Prostanoids, including PGs, such as PGD_2_, PGE_2_, PGI_2_, and PGF_2α_, represent, to date, a central subject of study among eicosanoids, especially in light of the ability of non-steroidal anti-inflammatory drugs (NSAIDs) to block their synthesis by covalent inhibition of COX-1/2 (19), which in turn results in the forestall of inflammation. The fact that NSAIDs are mostly used to treat acute inflammatory symptoms, such as swelling and pain, while being essentially ineffective on chronic conditions (for which steroidal drugs are preferred as treatment), has led to the idea that prostanoids are far less involved in chronic inflammatory pathologies (13). However, recent studies conducted using knockout mice for each specific GPR of the different classical eicosanoids (e.g., EP1–4, DP1–2, IP, FP, and TP), or specific stimulation by means of selective agonists, unveiled that their role might go well beyond the acute inflammatory response. Indeed, PG signaling—especially the one mediated by PGE_2_ and PGI_2_—seems to be involved in the sustained inflammation that causes the transition to chronic inflammation by acting as “cytokine amplifiers” (12, 20). These observations were based on animal and cellular models of chronic inflammatory diseases, such as arthritis (21) and cancer (22), where PGs are known to be involved also in their pathogenesis. In general, PGs induce chronic inflammation through five main mechanisms: (i) enhancement of the pro-inflammatory cytokines release cascade (21); (ii) amplification of innate immunity response to pathogen- and damage-associated molecular patterns (PAMPs and DAMPs) (23); (iii) activation of specific pro-inflammatory subsets of T helper cells, e.g., T_H_1 and T_H_17 (24, 25); (iv) recruitment of immune cells associated with chronic inflammation (e.g., macrophages, T and B cells) by synergistically acting with chemokines (12); (v) increase of pro-inflammatory genes induced by cytokines. Consistently, many studies have reported associations between specific PG-related genes (e.g., biosynthesizing enzymes or receptors) and the susceptibility to several chronic diseases, including Crohn’s disease (CD) (26), asthma (27), and multiple sclerosis (MS) (28, 29).

The main role of LTs in acute inflammation is to induce, alongside prostanoids, edema, and neutrophil influx within inflamed tissues (13). However, LTs are also central in perpetuation of inflammatory signals that lead to tissue damage in many chronic diseases. Indeed, LTs and their cysteinyl derivatives have been long known to be intimately connected to the pathogenesis of atherosclerosis, inflammatory bowel disease (IBD), psoriasis, rheumatoid arthritis (RA), as well as bronchial asthma, and MS, acting as chemoattractants for neutrophils, macrophages, eosinophils, and also T_H_17 lymphocytes (30–32), thus maintaining an ongoing and sustained inflammatory milieu. Of note, discussing the precise role of each LOX-derived eicosanoid in chronic inflammation is not an easy task, mainly due to their vast number (over 70 mediators), their differential action on cellular targets, and their complex and intermingled metabolic destiny. For instance, 5(S)-HpETE is the precursor of LTA_4_, which in turn is the common precursor of all bioactive leukotrienes (33), and of LX, which instead are anti-inflammatory inasmuch as are involved in the resolution of inflammation, as discussed in the next section.

## Specialized Pro-resolving Lipid Mediators

**Figure 1:**
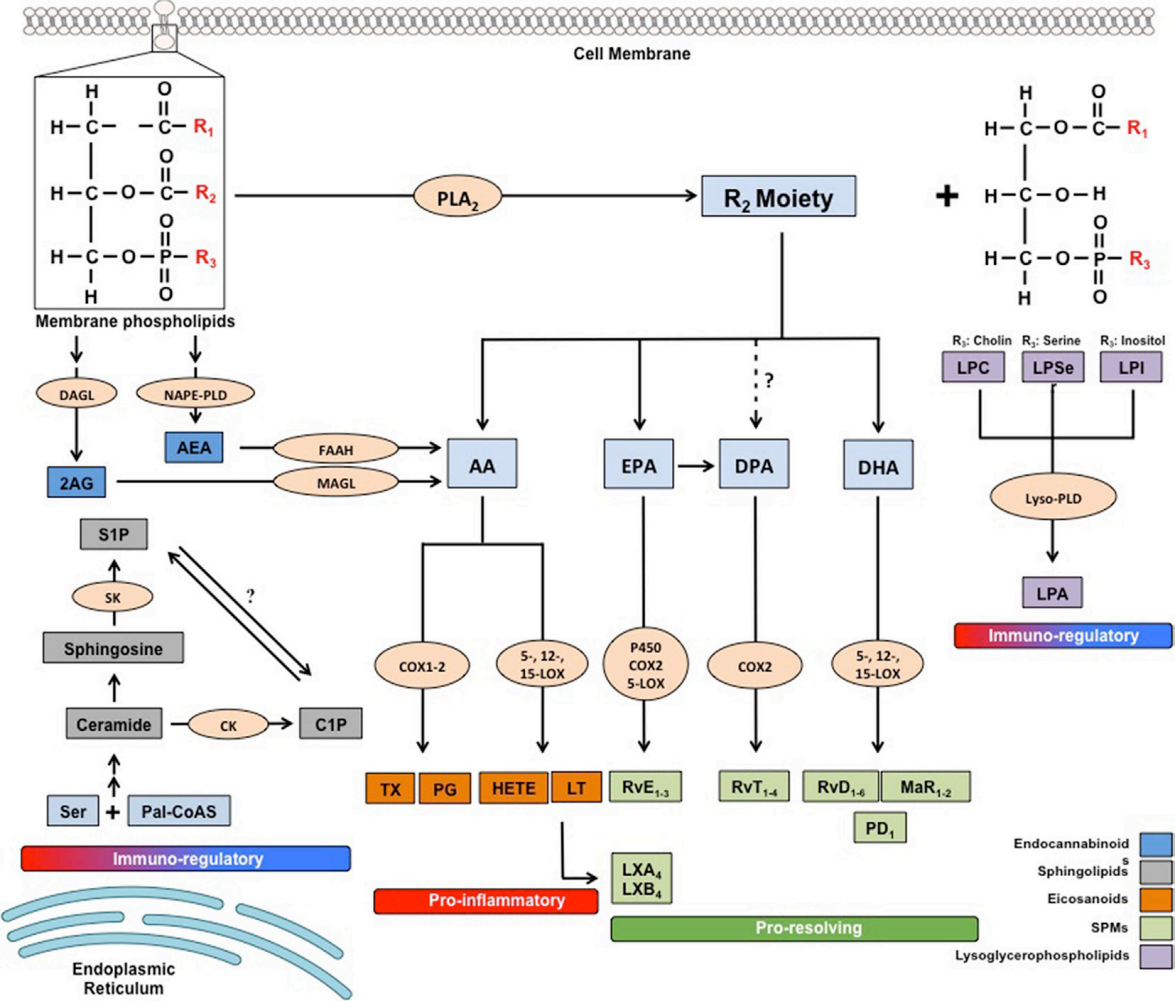
Metabolic pathways of the main families of endogenous bioactive lipids. 2-AG, 2 arachidonoylglycerol; AA, arachidonic acid; AEA, arachidonoylethanolamide; C1P, ceramide-1-phosphate; CK, ceramide kinase; COX, cyclooxygenase; DAGL, diacylglycerol lipase; DHA, docosahexaenoic acid; DPA, docosapentaenoic acid; EPA, eicosapentaenoic acid; FAAH, fatty acid amide hydrolase; HETEs, hydroxyeicosatetraenoic acids; LOX, lipoxygenase; LPA, lysophosphatidic acid; LPC, lysophosphatidilcholine; LPI, lysophosphatidylinositol; LPSer, lysophosphatidylserine; LTs, leukotrienes; LX, lipoxin; Lyso-PLD, lyso-phospholipase D; MAGL, monoacylglycerol lipase; MaR, maresin; NAPE-PLD, *N*-arachidonoylphosphatidylethanolamide-specific phospholipase D; Pal-CoASH, palmitoyl coenzyme A; PD, protectin; PDX, protectin DX; PGs, prostaglandins; PLA_2_, phospholipase A_2_; Rv, resolvin; S1P, sphingosine-1-phosphate; SK, sphingosine kinase; TXs, thromboxanes.

As mentioned above, at the peak of acute inflammation the very same cells involved in the production of pro-inflammatory lipid mediators undergo a class switch and start producing SPMs from ω-6 AA and even more from ω-3 PUFAs eicosapentaenoic acid (EPA), docosahexaenoic acid (DHA), and docosapentaenoic acid (DPA), through the stereoselective and concerted action of the same enzymes engaged in classical eicosanoids production: COXs, LOXs, and P450. To date, more than 20 different SPMs have been identified *via* sophisticated lipidomic approaches in the laboratory of Prof. Serhan, and these can be generally subdivided into six main classes: AA-derived LXs (LXA_4_ and LXB_4_); EPA-derived E-series resolvins (RvE_1–3_); DHA-derived D-series resolvins (RvD_1–6_); protectins/neuroprotectins (PD1/NPD1 and PDX); and their sulfido-conjugates (PCTRs), maresins (MaR1 and MaR2); and their conjugates (MCTR1–3), as well as the latest class to be identified, namely the DPA-derived 13-series resolvins (RvT_1–4_) (6, 34, 35) (Figure 1 summarizes the details of their respective biochemical synthesis). The lipid class switch is initiated already in the early phases of inflammation by LXA_4_ and LXB_4_, produced by platelets that progressively aggregate at the sites of inflammation (36). Overall, SPMs act as “immunoresolvents,” that is immune-pharmacological agents of resolution, as opposed to immunosuppressive agents, and they induce cessation of further leukocyte infiltration, recruitment and stimulation of nonphlogistic mononuclear cells, promote killing and clearance of pathogens and macrophage-mediated phagocytosis of apoptotic granulocytes (efferocytosis) and cellular debris, inhibit proinflammatory cytokines while inducing the production of anti-inflammatory mediators, shorten the time of resolution and activation of endogenous resolution programs, and promote tissue regeneration and healing (6, 37). Their activity is mediated by five separate GPRs, namely the formyl peptide receptor 2 (FPR2, also known as ALX), GPR32 (or DRV1), chemerin receptor 23 ChemR23 (or ERV), leukotriene B_4_ receptor 1 (BLT1) and GPR18 (or DRV2), expressed in different cell tissues and with differential affinities for each SPM or other lipid mediators (38). The target receptors of most SPMs are yet to be identified and further studies will be required to characterize the signaling pathways underlying their functions.

Although most of the insight gathered so far on SPMs concerns their role in modulating acute inflammation innate components, recent investigations have reported their ability to directly modulate adaptive immune cells, such as B and T lymphocytes, which are strongly involved in chronic detrimental inflammation. Indeed, although only RvD1 has been shown to act on B cells by inducing differentiation into plasma cells and promoting IgM and IgG antibody isotype switching (39) while inhibiting IgE production (40), a growing number of studies are now reporting direct or indirect effects of several SPMs on T cells. For instance, LXA_4_ and LXB_4_ both inhibited TNF-α secretion from activated human T cells (41), whereby the LXA_4_-induced effects were dependent on FPR2/ALX, which is expressed on T cells and their subsets (41–43). Additionally, RvE1, RvD1, and PD1 all have been shown to reduce the recruitment of CD4^+^ and CD8^+^ T cells (44–47), with the former SPM also limiting CD4-associated production of IFN-γ and IL-4 (45).

Of note, our group recently demonstrated that RvD1, RvD2, and MaR1 are able to hinder the production of pro-inflammatory cytokines in CD4 and CD8 T cells, as well as to inhibit *de novo* differentiation into T_H_1 and T_H_17, while promoting development of Treg cells without exerting any immunosuppressive and cytotoxic effect (43). Moreover, mice genetically unable to produce DHA displayed an increase in T_H_1/T_H_17 cells and a decrease in Treg cells (43), implying that SPMs impact on the balance between pathogenic and tolerogenic adaptive immune cells. Taken together, these findings support the view that SPMs may prevent chronicity of inflammation and/or autoimmunity and link resolution to adaptive immune cell responses. Interestingly, recent evidence indicates that pathologic conditions associated with altered SPM metabolism and function can contribute to chronicity and magnitude of persistent inflammatory conditions; as a result current research is centered on investigating the role of SPMs in chronic diseases in several mouse models and humans. Accordingly, decreased production of LX, E-, and D-series resolvins in the airways, as well as disruption of FPR2/ALX signaling have been linked to the pathogenesis of chronic obstructive pulmonary disease (COPD), and their restoration determined beneficial effects (48). Dysfunctional production of D-series resolvins and insufficient resolution has also been observed in mouse models or in human plasma samples of other typical chronic inflammatory and/or autoimmune diseases, such as (i) type-2 diabetes and obesity (49, 50); (ii) RA, in which low levels of RvD3 are associated with delayed resolution in mice and active disease in humans (51) and where RvD1 exerts protective actions on cartilage of murine model of inflammatory arthritis (52); (iii) atherosclerosis, in which evidence for impairment of resolution of vascular inflammation is governed by specific SPMs (53) and reduced RvD1 correlates with atherosclerotic plaque instability (30), and where RvD2, RvE1 and MaR1 have all been reported to have atheroprotective effects (54–57); and (iv) inflammatory bowel disease (58, 59). Accumulating evidence reveals that several neurodegenerative diseases characterized by chronic inflammation, such as MS, Alzheimer’s disease (AD) and amyotrophic lateral sclerosis (ALS), also seem to be associated to failure of activating pro-resolving mechanisms, and interventions with SPMs *in vitro* or *in vivo* exert neuroprotective properties. Indeed, a dysfunctional resolution pathway in SPMs and their receptors is present in post-mortem tissues of AD patients (60, 61) and several SPMs promoted neuronal survival and β-amyloid uptake by microglia *in vitro* (61–64). Additionally, RvD1 strongly inhibited cytokine release from inflammatory macrophages in ALS spinal cord (65), and its daily administration in mouse models of MS decreased disease progression by suppressing autoreactive T cells and by inducing an M2 phenotype of monocytes/macrophages and resident brain microglial cells (66). These findings advocate the stimulation of resolution pathways as a new therapeutic strategy to prevent chronic inflammation. Topical formulation of analogs of resolvin E1 (RX-10001, RX-10008, and RX-10045) or neuroprotectin D1 (RX-20001), resistant to metabolic inactivation, are currently underway in a number of human clinical trials for several chronic conditions, such as dry eye, macular degeneration and diabetic retinopathy, as well as lung, gut and kidney inflammation (67).

## Lysophospholipids and Sphingolipids

These bioactive lipids comprise many compounds asymmetrically distributed in plasma membranes with glycerol or sphingosine as respective backbones, and are characterized by a great molecular diversity due to their linkage with other molecules, such as ethanolamine, choline, inositol, serine or and fatty acids (e.g., phosphoinositides, lysoglycerophospholipids, and ceramides). The detailed metabolic steps of these substances are illustrated in Figure 1. Biochemical interconversions between them and also into other classes of bioactive lipids, such as eicosanoids and eCBs, are also possible, thanks to the action of phospholipases, lipid kinases and lipid-phosphate phosphatases (68, 69). The most biologically active lysophospholipids, derived from membrane phospholipids by removal of one or both fatty acids, are lysophosphaditylcholine (LPC) and lysophosphatidilinositol (LPI), and their byproduct lysophosphatidic acid (LPA), which are signaling molecules involved in pivotal aspects of cellular and tissue biology, such as plasma membrane shaping (70), cell growth and death (71), and inflammatory cascades (72). LPC and LPA modulate immune responses mostly by controlling distribution, trafficking and activation of immune cells (72–75), and their sustained activation have been suggested to be linked with several chronic inflammatory diseases, including obesity and diabetes (76, 77), cancer (74), atherosclerosis (78) and RA (79, 80).

On the other hand, the main active sphingolipids, whose peculiar chemical structure has baffled scientists for a long time (hence their name, inspired by the Egyptian Sphynx) (81), are ceramide and their byproducts ceramide 1-phosphate (C1P) and sphingosine 1-phosphate (S1P), shown in Figure 1 (69). Sphingolipids participate in numerous inflammatory processes and are responsible for controlling intracellular trafficking and signaling, cell growth, adhesion, vascularization, survival, and apoptosis (9, 68, 82), even though specific receptors have only been identified for S1P. The role of these three sphingolipids in chronic inflammation has been extensively investigated in the past decade, and have been mostly associated with immune-dependent and vascular-related chronic inflammatory diseases, including diabetes and obesity, COPD, IBD and neuroinflammatory disorders. For instance, an excessive ceramide signaling determines adipose tissue inflammation and insulin resistance, leading to obesity and type-2 diabetes, by inducing overactive immune cells like macrophages and B cells (81–83). Of note, most of the pro-inflammatory activities of ceramide seem to be mediated through its C1P and S1P metabolites. The former enhances both acute and chronic inflammatory responses by promoting phospholipase A2-mediated eicosanoid storm and by inducing cytokine production (84, 85). However, ceramide and C1P have also been shown to negatively regulate some pro-inflammatory cytokines (86, 87), suggesting a more complex role for them in inflammation. Furthermore, C1P has also been reported to impact on insulin resistance-induced type-2 diabetes and metabolic syndrome (82), as well as to induce cell migration in several cellular models of monocytes/macrophages and endothelial cells, as reviewed in Ref. (82), implying that this bioactive lipid might be involved in chronic inflammatory diseases characterized by migration of immune cells to inappropriate sites, such as IBD, atherosclerosis, and MS. Ceramide and its metabolites are also involved in the physiological regulation of endothelial/vascular integrity and function, whereby alterations of these sphingolipids are associated with vascular dysfunctions, and thus with chronic inflammatory states (88, 89).

Sphingosine-1-phosphate is arguably the best-studied molecule of this family of bioactive lipids and its actions are mediated by five identified receptors (S1PR1–5) (90). This lipid is a key mediator for lymphocyte trafficking between lymphoid and non-lymphoid tissues, favoring the egress of effector T and B cells from lymph nodes, thymus, bone marrow, and spleen, and blocking the ability of immature dendritic cells to migrate (91, 92). This function of S1P is particularly important, since T and B cells are the fire-starters of many (if not all) chronic inflammatory conditions and autoimmune diseases, in which modulation of their function is often exploited to develop new therapeutic strategies. Accordingly, the commercially available oral drug Fingolimod was developed as a first-line disease-modifying treatment for MS due to its ability to downregulate S1PR1, and hence to sequester highly pathogenic T cells (i.e., Th1 and Th17 cells) within the lymph nodes, avoiding brain invasion and myelin damage (93, 94). Fingolimod has also been shown to reduce blood–brain barrier dysfunction, a renowned pathogenetic mechanism of MS, by attenuating the production of sphingolipids from reactive astrocytes, including ceramide (95), also because several S1P receptors are significantly upregulated in MS lesions (96, 97). Interestingly, high S1P levels have also been found in patients with IBD and asthma (81, 98) and, accordingly, these conditions were attenuated by genetic deletion of the enzyme responsible for its synthesis in rodent models of disease or by pharmacological modulation of the S1P–S1PR axis (98–100).

## Endocannabinoids

Endocannabinoids include a group of bioactive lipids endogenously produced by humans and animals that are able (although with different affinities) to bind to and activate the same receptors as the main psychoactive component of marijuana Δ^9^-tetrahydrocannabinol, i.e., type-1 and type-2 cannabinoid receptors (CB_1_ and CB_2_). Arachidonoylethanolamide (commonly known as anandamide, AEA) and 2-arachidonoylglycerol (2-AG), both identified in the early 1990s, are the two best studied members of the eCB family, which also comprise 2-AG-ether, *O*-arachidonoylethanolamine, and palmitoylethanolamide (PEA) (10, 101). These molecules are ubiquitously produced by most tissues and immune cells, which are fully capable to metabolize them *via* a set of specific synthesizing [*N*-acyl-phosphatidylethanolamine-hydrolyzing phospholipase D (NAPE-PLD) for AEA and its congeners and diacylglycerol lipase (DAGL) for 2-AG] and degrading [fatty acid amide hydrolase FAAH for AEA and monoacylglycerol lipase (MAGL) for 2-AG] enzymes (101). Besides the aforementioned CB_1_ and CB_2_ receptors, eCBs also engage other molecular targets that include members of the transient receptor potential (TRP) channels, GPR55, and peroxisome proliferator-activated receptors (PPARs), differentially expressed by body districts also according to their inflammatory state (102, 103). Altogether, eCBs and their enzymes and receptors constitute the so-called “eCB system,” which generally serves as a homeostatic system that controls several physiopathological states ultimately maintaining human health (104). In particular, eCBs are arguably among the most potent immunoregulatory compounds, capable of regulating the functions of several cell subsets of either innate or adaptive immunity (in particular monocytes/macrophages, dendritic cells, granulocytes, and T lymphocytes), with AEA and PEA being mostly anti-inflammatory (105–107) and 2-AG both pro- and anti-inflammatory (10, 108–111). Indeed, due to their role in the overall control of tissue homeostasis, variations in the tone of distinct eCBs within tissues, or in the expression of their metabolic enzymes or receptors have been clearly recognized as central in the pathophysiology of many chronic inflammatory diseases. Indeed, it is now clear that perturbations in all members of the eCB system occur during every chronic inflammatory process, from cancer, metabolic, and gastrointestinal diseases to autoimmune and neuroinflammatory disorders [extensively reviewed in Ref. (112–119)]. This is because every single cell and tissue of our body produces specific eCBs (sometimes also simultaneously) “on demand” and at certain concentrations according to the stimulus and/or the need, in order to autocrinally or paracrinally orchestrate the inflammatory responses of nearby cells through complex interactions between multiple receptors or targets with different but partly overlapping activities (104). Recently, eCBs are also emerging as pro-resolving agents due to the ability of AEA and 2-AG, alongside other congeners (e.g., PEA) to boost resolution programs during neuroinflammation (120, 121), while 2-AG alone can enhance phagocytosis in human macrophages (122). Accordingly, several experimental models of chronic inflammatory diseases have been instrumental, not only to better understand the role of each member of the eCB system in their different pathogenic mechanisms, but mostly to identify in the pharmacological manipulation of either receptors or enzymes (by means of selective activation of specific receptors or inhibition of AEA, 2-AG, or PEA degrading enzymes) a promising therapeutic strategy. In line with this, modulation of the eCB system has been shown to be beneficial by attenuating inflammatory processes that include cytokine release, infiltration of leukocytes at inflamed sites, production of reactive oxygen and nitrogen species, and overall immune cell activation (123–127). These effects were particularly relevant in several neuroinflammatory and neurodegenerative diseases, such as MS and AD (119, 128–131), where chronic inflammation is indeed a hallmark and whereby targeting the eCB system seems to be a promising therapeutic approach in the near future. Interestingly, AEA and 2-AG can also be metabolized by COX-2, LOXs, and P450 into eicosanoid-like PG-ethanolamides and glyceryl esters, hydroxy-anandamides, and hydroxyeicosatetraenoyl-glycerols, respectively (132, 133). The function and biological activity of these lipids is still unclear, but it is plausible that they might play a role in chronic inflammation.

## Do Bioactive Lipids Coexist during the Different Stages of Inflammation?

The array of bioactive lipids that lays at the heart of tissue immune homeostasis represents a vastly intertwined network of molecules whose metabolism is rather complex, in that not only they undergo fast biosynthesis, degradation or interconversion, but also they share common metabolic enzymes, the activation or regulation of which is fascinating and still not completely unraveled. Consequently, the full elucidation of their temporal production and their role in the different phases or inflammation (from acute inflammation and its resolution to chronic inflammation) represents conceivably one of the biggest challenges of our time. To date, this has been particularly investigated for eicosanoids and SPMs in terms of their detailed temporal and spatial production and their specific role during the different inflammatory states and this is mainly due to their thorough characterization by means of lipidomics analyses performed locally in inflammatory or self-resolving tissues (i.e., edema). Indeed, both eicosanoids and SPMs are present in all phases of inflammation: the former are massively produced within the first 2–4 h and then show a reduction during the resolution phase, whereas the latter appear already at early phases of inflammation (especially LX and RvTs), usually reach their highest level at the peak of acute inflammation (6–12 h) and some specific molecules (i.e., RvD3) are produced at later stages. During chronic inflammation, as previously described, both families of bioactive lipids are present, with specific molecules being overly or inadequately produced, according to the different inflammatory diseases and tissues. Such temporal and spatial production, although less studied, is beginning to hold true also for the other families of bioactive lipids. Indeed, eCB levels raise rapidly following noxious stimuli and this is generally associated with their role in activating anti-inflammatory and protective mechanisms, although persistent inflammation usually dysregulates the eCB system in a way that their action might even become detrimental (134). On the other hand, other authors have reported diminished levels of eCBs in chronic inflammatory models, even after several days after the induction of inflammation (135). Of note, the temporal production of eCBs can only be inferred from *in vitro* studies conducted on cell lines activated with different inflammatory stimuli at different times or in tissues of patients or animal models of acute or chronic inflammatory diseases. To date, a fully detailed temporal characterization of each eCB during the different stages of inflammation, namely from an acute model of inflammation to a *bona fide* model of spontaneous resolution, is still absent and represents a future challenge. This scenario is further complicated by the fact that eCBs exist in dynamic equilibria with different other lipid-derived mediators, including eicosanoids, prostamides, and their recently identified ω-3 congeners (136).

Also sphingolipids are found during different stages of inflammation, and are likely part of the resolution machinery as suggested by the fact that apoptotic cells at the inflammation sites attract pro-resolving macrophages in a S1P-S1PR1-dependent manner (137), while neutrophil apoptosis, which is pivotal in initiating resolution, rely at least in part on the generation of ceramide (138). Furthermore, LPA might represent another brick in the resolution wall, in that it has been recently reported to be rapidly produced during the resolution phase of tissue inflammation and to recruit monocytes *via* the common pro-resolving receptor ALX/FPR2 (139). Interestingly, treatment of human fibroblasts (key cells involved in tissue healing and regeneration) with TNF-α, a cytokine that is mainly produced during acute or chronic inflammation, results in a significant increase in S1P levels, which rapidly returns to baseline within less than an hour (140), and of COX-2 expression, which can, in turn, temporally generate both eicosanoids, SPMs or even eCBs metabolites.

All these evidences not only account for a coexistence of several families of bioactive lipids during the different stages of inflammation, but also suggest that each inflammatory phase requires the concerted action of such lipid mediators, which are also likely to molecularly interact and engage in physiopathological cross talks.

## Concluding Remarks

For a long time, the idea that lipids were mere constituents of cellular membranes and efficient energy sources was indisputable, but over the past two decades, not only it became clear that they actually harbor many functions in the regulation of intercellular and intracellular signaling pathways, but also that they represent bioactive molecules that are able to orchestrate a plethora of biological activities on their own, in order to maintain tissue homeostasis by governing body’s defensive and healing processes like inflammation and its resolution. During these processes, several families of bioactive lipids are temporally and spatially engaged so that the appropriate leukocytes are recruited and the noxious agent or stimulus is eliminated. Accordingly, classical eicosanoids are the fire-starters of the inflammatory processes and engage mainly cells of the innate arm of immunity that execute all possible strategies to quickly eradicate the injury. If the danger ceases or is successfully terminated, the fire of inflammation is elegantly extinguished by SPMs that recruit non-phlogistic innate immune cells and activate resolution pathways, aimed at healing the damaged tissue. On the contrary, if the injurious stimulus is either persistent or not eliminated, perhaps by failure of resolving inflammation, a wildfire of long-lasting inflammatory processes occurs and the flame of inflammation is kept alive mainly by cells of the adaptive arm of immunity, thus leading to many chronic inflammatory diseases. Under these circumstances, tissues activate several adaptation mechanisms that allow cells to cope with the changes induced by the damage, also thanks to other families of bioactive lipids like lysophospholipids, sphingolipids, and eCBs, that ultimately regulate cell growth, differentiation, and destiny with the goal of helping the body to restore homeostatic balance. Most of these bioactive lipids and several elements of their complex metabolism and signaling (i.e., enzymes and receptors) are differentially dysregulated in many chronic inflammatory diseases (Table 1), suggesting that managing the fire within by targeting the endogenous mechanisms involved in the spontaneous fire extinction, or in the modulation of homeostatic processes, rather than simply suppressing inflammation, could be a future and promising therapeutic strategy to be undertaken.

**Table 1:** Main role of bioactive lipids in chronic inflammatory diseases.

Bioactive lipid family	Bioactive lipid	Chronic inflammatory diseases	Effect
Classical eicosanoids	PGE_2_, PGI_2_	Rheumatoid arthritis (RA)	Cytokine amplification, enhanced innate immune responses, and recruitment of adaptive immune cells
		Cancer	
		Crohn’s disease	
		Asthma	
		Multiple sclerosis (MS)	

	LTB_4_	Psoriasis	Leukocyte chemotaxis and trafficking
		RA	
		Asthma	
		Inflammatory bowel disease (IBD)	
		Atherosclerosis	

Specialized pro-resolving mediators (SPMs)	LXA_4_	Alzheimer’s disease (AD)	Decreased production and neuroprotective effects

	RvD1	Chronic obstructive pulmonary disease	Decreased production and beneficial effects
		Atherosclerosis	Impaired metabolism and correlation with plaque instability
		Obesity/type-2 diabetes	Decrease adipose tissue and improvement of insulin sensitivity
		RA	Protective on cartilage
		AD	Correlation with cognitive functions and β-amyloid phagocytosis
		MS	Th1/Th17 suppression and M2 induction
		Amyotrophic lateral sclerosis (ALS)	M1 macrophages suppression

	RvD2	Obesity/type-2 diabetes	Decreased production
		Atherosclerosis	Atheroprotective effects

	RvD3	RA	Decreased production

	PDX	AD	Decreased production and neuroprotective effects

	MaR1	AD	Decreased production and β-amyloid phagocytosis

Lysoglycero-phospholipids	Lysophosphatidic acid (LPC), lipoxygenase (LPA)	Obesity/type-2 diabetes	Sustained production and promotion of inflammatory cascades
		Cancer	
		Atherosclerosis	
		RA	

Sphingolipids	Ceramide, sphingosine-1-phosphate (S1P), ceramide-1-phosphate	Type-2 diabetes	Adipose tissue inflammation, insulin resistance, and activation of immune cells
		Atherosclerosis	Leukocyte recruitment and vascular dysfunction
		IBD	

	S1P	MS	Signaling disruption and trafficking of T and B cells from lymphoid organs
		IBD	Increased levels and beneficial effects
		Asthma	

Endocannabinoids (eCBs)	Arachidonoylethanolamide, 2-arachidonoylglycerol	Cancer	Differential alteration of their levels and beneficial effects when administered or upon genetical/pharmacological manipulation of a member of the eCB system
		Metabolic diseases	
		Gastrointestinal diseases	
		Atherosclerosis	
		Autoimmune diseases	
		MS	
		AD	
		PD	
		ALS	
		Mood disorders	

	Palmitoylethanolamide	Chronic granulomatous inflammation	Decreased levels and beneficial effects
		MS	Increased levels and reduction of motor disability in animal models
		Neuropathic pain	Anti-allodynic and anti-hyperalgesic effects *via* modulation of microglial and mast cell activity

## Author Contributions

All authors listed have made a substantial, direct, and intellectual contribution to the work and approved it for publication.

## Conflict of Interest Statement

The authors declare that the research was conducted in the absence of any commercial or financial relationships that could be construed as a potential conflict of interest.
